# Supplementary material for: Treatment of stimulant use disorder: A systematic review of reviews
Source: PLoS One. 2020 Jun 18;15(6):e0234809. doi: 10.1371/journal.pone.0234809 (PMC7302911; doi:10.1371/journal.pone.0234809)
Supplement: S2 Appendix — (DOCX) [file pone.0234809.s002.docx]

**Appendix S2: Search strategy**

I: Pubmed

*Search 1:* Cocaine dependence AND treatment AND (systematic review OR meta-analysis)

("cocaine-related disorders"[MeSH Terms] OR ("cocaine-related"[All Fields] AND "disorders"[All Fields]) OR "cocaine-related disorders"[All Fields] OR ("cocaine"[All Fields] AND "dependence"[All Fields]) OR "cocaine dependence"[All Fields]) AND ("therapy"[Subheading] OR "therapy"[All Fields] OR "treatment"[All Fields] OR "therapeutics"[MeSH Terms] OR "therapeutics"[All Fields]) AND ("systematic review"[All Fields] OR "meta-analysis"[All Fields]))

*Article type: Systematic review, Meta-analysis*

**Results: 78**

*Search 2:* Stimulant dependence AND treatment AND (systematic review OR meta-analysis)

(("central nervous system stimulants"[Pharmacological Action] OR "central nervous system

stimulants"[MeSH Terms] OR ("central"[All Fields] AND "nervous"[All Fields] AND "system"[All Fields] AND "stimulants"[All Fields]) OR "central nervous system stimulants"[All Fields] OR "stimulant"[All Fields]) AND ("dependency (psychology)"[MeSH Terms] OR ("dependency"[All Fields] AND "(psychology)"[All Fields]) OR "dependency (psychology)"[All Fields] OR "dependence"[All Fields])) AND ("therapy"[Subheading] OR "therapy"[All Fields] OR "treatment"[All Fields] OR "therapeutics"[MeSH Terms] OR "therapeutics"[All Fields]) AND (("systematic review"[All Fields]) OR "meta-analysis"[All Fields]))

*Article type: Systematic review, Meta-analysis*

**Results: 18**

*Search 3:* Stimulant use disorder AND treatment AND (systematic review OR meta-analysis)

(("central nervous system stimulants"[Pharmacological Action] OR "central nervous system stimulants"[MeSH Terms] OR ("central"[All Fields] AND "nervous"[All Fields] AND "system"[All Fields] AND "stimulants"[All Fields]) OR "central nervous system stimulants"[All Fields] OR "stimulant"[All Fields]) AND ("disease"[MeSH Terms] OR "disease"[All Fields] OR "disorder"[All Fields])) AND ("therapy"[Subheading] OR "therapy"[All Fields] OR "treatment"[All Fields] OR "therapeutics"[MeSH Terms] OR "therapeutics"[All Fields]) AND (("systematic review"[All Fields]) OR "meta-analysis"[All Fields]))

*Article type: Systematic review, Meta-analysis*

**Results: 271**

*Search 4:* Methamphetamine dependence AND treatment AND (systematic review OR meta-analysis)

(("methamphetamine"[MeSH Terms] OR "methamphetamine"[All Fields]) AND ("dependency (psychology)"[MeSH Terms] OR ("dependency"[All Fields] AND "(psychology)"[All Fields]) OR "dependency (psychology)"[All Fields] OR "dependence"[All Fields])) AND ("therapy"[Subheading] OR "therapy"[All Fields] OR "treatment"[All Fields] OR "therapeutics"[MeSH Terms] OR "therapeutics"[All Fields]) AND (("systematic review"[All Fields]) OR "meta-analysis"[All Fields]))

*Article type: Systematic review, Meta-analysis*

**Results: 8**

**Total from Pubmed = 354**

EmBase (OVID):

1. Cocaine dependence/dt. rh. th [Drug Therapy, Rehabilitation, Therapy]
2. Amphetamine/or methamphetamine/
3. Drug dependence treatment/
4. “systematic review”
5. “meta-analysis”

(1 or 2) and 3 and (4 or 5)

**Results: 91**

**Total Results = 476**

**With duplicates removed = 453**

**Screened by title = 56** (397 excluded)

1. Not English (n=7)
2. Withdrawn (n=8)
3. Not focused on treatment of cocaine, amphetamine or stimulant dependence (n=382)

**Screened by abstract = 26** (30 excluded)

1. Not focused on treatment of stimulant use disorder: 14
2. Not systematic: 9
3. Updated review available: 7

**Additional reviews screened by hand = 3**

**Total included reviews = 29**
