# Supplementary material for: Treatment of stimulant use disorder: A systematic review of reviews
Source: PLoS One. 2020 Jun 18;15(6):e0234809. doi: 10.1371/journal.pone.0234809 (PMC7302911; doi:10.1371/journal.pone.0234809)
Supplement: S3 Appendix — (DOCX) [file pone.0234809.s003.docx]

Appendix S3: Complete list of pharmaceutical interventions assessed in this review.

| **Intervention** | **Medication** | **# of Trials** |
| --- | --- | --- |
| ***Antidepressants:*** |  |  |
| *Pani et al. 2011* | Desipramine | 17 |
|  | Fluoxetine | 5 |
|  | Amantadine | 3 |
|  | Bupropion | 3 |
|  | Nefazodone | 2 |
|  | Ritanserin | 2 |
|  | Buspirone | 1 |
|  | Carbamazepine | 1 |
|  | Citalopram | 1 |
|  | Doepezil | 1 |
|  | Gepirone | 1 |
|  | Imipramine | 1 |
|  | Lithium Carbonate | 1 |
|  | Paroxetine | 1 |
|  | Pentoxifylline | 1 |
|  | Pramipexole | 1 |
|  | Riluzole | 1 |
|  | Selegiline | 1 |
|  | Sertraline | 1 |
|  | Tigabine | 1 |
|  | Tryptophan | 1 |
|  | Venlafaxine | 1 |
| *Chan et al. 2019^1,2^* | Fluoxetine | NR |
|  | Paroxetine | NR |
|  | Sertraline | NR |
|  | Desipramine | NR |
|  | Bupropion | NR |
|  | Mirtazapine | NR |
|  | Nefazodone | NR |
|  | Venlafaxine | NR |
| *Torrens et al. 2005* | Desipramine | 9 |
|  | Fluoxetine | 8 |
|  | Imipramine | 2 |
|  | Amantadine | 1 |
|  | Bupropion | 1 |
|  | Gepirone | 1 |
|  | Ritanserine | 1 |
| *Chan et al. 2019^3^* | Bupropion | 1 |
|  | Mirtazapine | 1 |
|  | Sertraline | 1 |
| **Disulfiram** |  |  |
| *Pani et al. 2010* | Disulfiram | 7 |
| **Dopamine Agonists** |  |  |
| *Minozzi et al. 2015* | Amantadine | 11 |
|  | L-dopa/Carbidopa | 6 |
|  | Bromocriptine | 5 |
|  | Pergolide | 2 |
|  | Pramipexole | 1 |
|  | Propanolol | 1 |
|  | Hydergine | 1 |
|  | Cabergoline | 1 |
| *Chan et al. 2019^1,2^* | Amantadine | NR |
|  | Bromocriptine | NR |
|  | Cabergoline | NR |
|  | Hydergine | NR |
|  | L-Dopa/Carbidopa | NR |
|  | Pergolide | NR |
|  | Pramipexole | NR |
| **Antipsychotics** |  |  |
| *Indave et al. 2016* | Olanzapine | 5 |
|  | Risperidone | 5 |
|  | Quetiapine | 2 |
|  | Aripiprazole | 1 |
|  | Haloperidol | 1 |
|  | Lamotrigine | 1 |
|  | Reserpine | 1 |
|  | Ropinirole | 1 |
| *Chan et al. 2019^1,2^* | Aripiprazole | NR |
|  | Haloperidol | NR |
|  | Lamotrigine | NR |
|  | Olanzapine | NR |
|  | Quetiapine | NR |
|  | Risperidone | NR |
|  | Reserpine | NR |
| *Alvarez 2013* | Risperidone | 7 |
|  | Olanzapine | 3 |
|  | Reserpine | 2 |
|  | Ritanserin | 2 |
|  | Quetiapine | 1 |
| *Chan et al. 2019^3^* | Aripiprazole | 2 |
| *Kishi et al. 2013* | Risperidone | 5 |
|  | Aripiprazole | 4 |
|  | Olanzapine | 3 |
|  | Reserpine | 2 |
| **Anticonvulsants** |  |  |
| *Minozzi et al. 2015* | Carbamazepine | 9 |
|  | Topiramate | 5 |
|  | Gabapentin | 3 |
|  | Tiagabine | 3 |
|  | Desipramine | 2 |
|  | Lamotrigine | 2 |
|  | Vigabatrin | 2 |
|  | Phenytoin | 1 |
| *Chan et al. 2019^1,2^* | Baclofen |  |
|  | Carbamazepine |  |
|  | Gabapentin |  |
|  | Lamotrigine |  |
|  | Phenytoin |  |
|  | Tiagabine |  |
|  | Vigabatrin |  |
|  | Topiramate | 5 |
| *Alvarez et al. 2010* | Carbamazepine | 7 |
|  | Tiagabine | 4 |
|  | Gabapentin | 2 |
|  | Lamotrigine | 1 |
|  | Phenytoin | 1 |
|  | Topiramate | 1 |
|  | Valproic Acid | 1 |
| *Chan et al. 2019^3^* | Gabapentin |  |
|  | Baclofen |  |
|  | Topiramate |  |
| *Singh et al. 2015* | Topiramate | 5 |
| **Psychostimulants** |  |  |
| *Castells et al. 2016* | Modafinil | 8 |
|  | Mazindol | 4 |
|  | Methylphenidate | 4 |
|  | Bupropion | 3 |
|  | Dexamphetamine | 2 |
|  | Dextroamphetamine | 2 |
|  | Lisdexamphetamine | 1 |
|  | Detroamphetamine | 1 |
|  | Methamphetamine | 1 |
|  | Mixed amphetamine salts | 1 |
|  | Selegiline | 1 |
| *Chan et al. 2019^1,2^* | Dexamphetamine | NR |
|  | Lisdexamfetamine | NR |
|  | Mazindol | NR |
|  | Methamphetamine | NR |
|  | Methyphenidate | NR |
|  | Mixed amphetamine salts | NR |
|  | Modafinil | NR |
|  | Selegiline | NR |
| *Perez-Mana et al. 2013* | Modafinil | 8 |
|  | Bupropion | 5 |
|  | Dexamphetamine | 2 |
| *Chan et al. 2019^3^* | Dexamphetamine | NR |
|  | Methylphenidate | 5 |
|  | Modafinil | NR |
| *Bhatt et al. 2016* | Modafinil | 3 |
|  | Bupropion | 6 |
|  | Dextroamphetamine | 1 |
|  | Methylphenidate | 6 |
|  | Dexamphetamine | 1 |
| *Perez-Mana et al. 2011* | Bupropion | 4 |
|  | Dexamphetamine | 2 |
|  | Disulfiram | 3 |
|  | Levodopa-carbidopa | 4 |
|  | Mazindol | 1 |
|  | Methamphetamine | 1 |
|  | Methylphenidate | 4 |
|  | Modafinil | 2 |
|  | Selegiline | 1 |
| *Sangroula et al. 2017* | Modafinil | 6 |
| *Dursteler et al. 2015* | Methylphenidate | 5 |
| **Opioid Agonists** |  |  |
| *Castells et al. 2009* | Buprenorphine | NR |
|  | Methadone | NR |
| **NAC** |  |  |
| *Echevarria et al. 2017* | NAC | 6 |

*^1^Chan et al. Pharmacotherapy for cocaine use disorder – a systematic review and meta-analysis ;^2^Results reported by outcome rather than by medication; ^3^Chan et al. Pharmacotherapy for methamphetamine/amphetamine use disorder – a systematic review and meta-analysis; NR, not reported; NAC, n-acetylcysteine.*
