# Supplementary material for: Treatment of stimulant use disorder: A systematic review of reviews
Source: PLoS One. 2020 Jun 18;15(6):e0234809. doi: 10.1371/journal.pone.0234809 (PMC7302911; doi:10.1371/journal.pone.0234809)
Supplement: S1 Table — (DOCX) [file pone.0234809.s004.docx]

*Table S1: Summary of population characteristics and key findings.*

| **Author** | **Population characteristics** | **Intervention (# Studies)** | **Inclusion/Exclusion Criteria** | **Main Findings** |
| --- | --- | --- | --- | --- |
| *De Crescenzo et al. 2018* | 50 RCTs, N = 6942  Mean age: 36.8  % Male: 64.1%  42 RCTs recruited from North America, 6 from Europe, 1 from Latin America, 1 from Oceania  76% of trials enrolled participants with cocaine use disorder, 8% amphetamine use disorder, 16% with both.  18 RCTs enrolled participants on methadone maintenance treatment. | CBT vs CM (4) CBT vs CM + CBT (6)  CBT vs non-contingent rewards (1)  CBT vs meditation based treatment (2)  CBT vs supportive-expressive psychodynamic therapy (1)  CBT vs TAU (7)  CM vs CM + CBT (5)  CM vs non-contingent rewards (8)  CM vs TAU (15)  CM + 12 step program vs CM + CRA (1)  CM + 12 step program vs 12-step program + non-contingent rewards (1)  CM + CRA vs CRA (1)  CM + CRA vs CRA (3)  CM + CRA vs CRA + non-contingent rewards (2)  CM + CRA vs 12-step program + non-contingent rewards (2)  CM + CRA vs TAU (1)  CM + 12-step program vs CRA + non-contingent rewards (1)  Meditation based treatments vs TAU (1)  Supportive-expressive psychodynamic therapy vs TAU (1)  12-step program vs CBT (3)  12-step program vs supportive-expressive psychodynamic therapy (1)  12-step program vs TAU (2)  12-step program + non-contingent rewards vs CRA + non-contingent rewards (1) | Randomized controlled trials comparing any psychosocial intervention against either TAU or another psychosocial intervention. Studies with comorbid substance use were included.  RCTs with less than 4 weeks duration, and studies on occasional users not actively seeking treatment were excluded. | Primary outcomes were proportion of individuals abstinent (determined by urinalysis) at the end of treatment, and participant dropout.  Secondary outcomes were abstinence at 12 weeks and at longest follow up, and longest duration of abstinence.  CM + CRA demonstrated the most significant benefit compared to CBT, 12-step programs and non-contingent rewards, and was associated with fewer dropouts. The benefit of CM + CRA persisted at longest follow up.  CM + CRA, CM + CBT, and CM alone increased abstinence at the end of treatment.  Subgroup analyses for abstinence and dropout did not differ substantially from primary results. Subgroups included: year of publications, sex ratio, mean age group, intensity of treatment, type of stimulant, risk of bias, opioid therapy, sample size, comorbid alcohol misuse. |
| *Schumacher et al. 2007* | 4 RCTs, N = 644  Age: around age 35  Participants are all homeless from Birmingham, Alabama  Primary crack cocaine addiction  Predominantly male | CM only vs. DT only vs. CM + DT vs. neither | 4 successive randomized clinical trials  Homeless persons with regular crack cocaine use | The primary outcome was drug abstinence, measured at 2 and 6-month assessment points. Significantly stronger benefit of CM + DT and CM alone compared to DT alone. |
| *Farronato et al. 2013* | 7 Studies, N = 972  Mean age: 32.7  Mean %male: 66.2  Participants with cocaine abuse or dependence or objective evidence of cocaine use in the prior 2 weeks.  Participants with concomitant substance use or nonsubstance comorbidities were included. | CM vs CBT vs CM + CBT vs social-support (control)  CBT vs CBT + CM  CM vs Treatment as usual  CM vs CBT vs CBT + CM  CM vs CBT vs CBT + CM vs control  Placebo + CBT vs Naltrexone + CBT vs Placebo + CBT + CM vs Naltrexone + CBT + CM  CBT vs CBT + CM comparing placebo vs levopoda in both arms | Only studies in which the interventions were validated or describe in enough detail to be replicated were included.  Studies in which pharmacological agents were administered in combination were also included. | Primary outcomes: cocaine abstinence, (determined by urinalysis, blood samples or self-report) + retention in treatment.  CM alone reliably reduced cocaine use during active treatment. Positive effects of CBT emerged after treatment. Additive effects were seen in 2 trials. |
| *Lee et al. 2008* | 8 studies assessed CM, N = 1,892  Participants with cocaine, Amphetamine and Methamphetamine dependence  3 studies: gay/bisexual men  1 study: enrolled in methadone maintenance | CBT vs CM vs CBT + CM (4)  Usual care vs Usual care + CM (3)  Sertraline only vs Sertraline + CM vs placebo + CM vs placebo only (1) | Trials were included that assessed efficacy of cognitive and behavioural therapies for stimulant dependence. | CM demonstrated a reduction of methamphetamine use during treatment, with unclear sustained effects at post-treatment follow-up. |
| *Schierenberg et al. 2012* | 26 studies, N = 1,742  Duration: range 12 wks- 1 yr  Participants with cocaine dependence, without comorbid opioid dependence or psychiatric diagnoses. | Standard treatment vs Standard treatment + CM (1)  CRA + CM vs Standard Treatment (3)  CRA with CM vs CRA only (3)  Contingent vs non-contingent incentives with standard treatment vs CRA (1)  CRA + CM vs CM only (1)  CM combined with dopaminergic agonists (2)  CM combined with Serotonin agents (3)  CM combined with Memantine (1)  CM combined with Naltrexone (1) | Studies were excluded when patients had comorbid opioid dependence, used methadone, were diagnosed with severe mental or neurological disorders or when studies were not RCTs. | Primary outcomes: retention in treatment and abstinence.  CM improved abstinence compared to standard treatment. CM + CRA is superior to standard treatment and to CRA alone in the outcomes of retention and abstinence. CM alone decreases post-treatment cocaine use but may be more effective when combined with CRA.  There is synergy between levodopa and CM with regards to abstinence. Antidepressants may become effective when combined with CM. |
| *Roozen et al. 2003* | 4 studies focused on cocaine dependence, N = 173  Age: mean 30.2 (range 18-65)  Duration: range 1 – 24 weeks | CRA + ‘incentives’ vs usual care (1)  CRA vs CRA + ‘incentives’ (1)  CRA + ‘incentives’ (independent of urine sample) vs CRA + ‘incentives’ (contingent on abstinence) (1)  CRA vs usual care (1) | RCTs with subjects whose substance dependence was not the main diagnosis, or whose addiction was not the main reason for contact, were excluded.  RCTs in which only one component of CRA was investigated were excluded.  RCTs in which pharmacological agents were used in combination with CRA were included. | Primary outcome: cocaine abstinence.  Significant increase in cocaine abstinence with incentives, when compared to usual care, and to CRA alone.  Effect was greater for longer treatment duration (4, 16 weeks vs <4 weeks) |
| *Harada et al. 2019* | 2 studies, N = 210  Mean age: 24.4  % Male: 68%  Participants were adults with amphetamine type stimulant dependence. Polysubstance use was common. | Single-session brief CBT vs waiting-list control (1)  Web-based CBT vs waiting-list control (1) | RCTs assessing CBT for the treatment of amphetamine type stimulant use disorder. Studies where CBT was assessed in conjunction with other psychotherapy interventions were excluded. | Primary outcomes: abstinence rate (measured by urine samples or self-reported), drug use, and dropout.  Secondary outcomes included overall mortality, psychological variables, and adverse outcomes.  Limited data available, with small sample sizes and significant participant dropout. |
| *Mills et al. 2005* | 9 studies, N = 1747  Participants with cocaine dependence.  1 study included only crack cocaine users.  3 studies only included patients using methadone in addition to cocaine. | Auricular acupuncture (9)  Auricular and body points acupuncture (2) | RCTs assessing acupuncture for treatment of cocaine dependence. Studies that included polysubstance users were excluded. | Primary outcome: cocaine abstinence.  No effect of acupuncture on cocaine abstinence. Average of 50% loss to follow up. |
| *Gates et al. 2008* | 7 studies, N = 1,433  People with cocaine or crack cocaine dependence.  One study excluded participants with polysubstance use. | Auricular acupuncture vs sham acupuncture (6)  Acupuncture vs no acupuncture (3) | RCTs comparing auricular acupuncture with sham acupuncture or no acupuncture | Primary outcome: attrition and cocaine use.  No differences between acupuncture and sham acupuncture or no acupuncture were found for attrition or cocaine use by any measure. |
|  |  |  |  |  |
| *Pani et al. 2011* | 37 studies, N = 2819  67% male  Mean age: 35 years  Participants with cocaine abuse and dependence  Mean duration of trials 10.7 weeks (range 2-25)  Participants with additional substance use diagnoses, and mental health comorbidities were included. | Antidepressants: (# trials)  Antidepressants vs placebo:  Desipramine (17)  Fluoxetine (5)  Bupropion (3)  Nefazodone (2)  Ritanserin (2)  Buspirone (1)  Gepirone (1)  Paroxetine (1)  Citalopram (1)  Venlafaxine (1)  Selegiline (1)  Tryptophan (1)  Sertraline (1)  Imipramine (1)  Other pharmacological interventions compared in the above trials:  Amantadine (3)  Carbamazepine (1)  Lithium carbonate (1)  Pentoxifylline (1)  Riluzole (1)  Pramipexole (1)  Donepezil (1)  Tiagabine (1) | All RCTs focused on antidepressant medication for cocaine dependence or problematic cocaine use were included  People under 18 years of age and pregnant women were excluded | Primary outcomes: Dropout, side effects, use of primary substance of abuse (self-reported or urine samples), cocaine use at follow-up.  No significant evidence of difference was found when comparing (1) antidepressants versus placebo or (2) antidepressants versus other drugs.  Subgroup analyses: scheduled length of trial, associated opioid dependence, associated psychosocial interventions.  Reduced dropout in trials with associated psychotherapy and those with associated opioid dependence. |
| *Chan et al. 2019* | 48 RCTs  66 different pharmacotherapy interventions were examined for cocaine use disorder.  Participants were adults with cocaine use disorder, without psychiatric comorbidities. | Antidepressants  Cognitive enhancing drugs (2)  Anxiolytics (1)  Anticonvulsants and muscle relaxants (23)  Topiramate (5)  Medications FDA Approved for other substance use disorders (Acamprosate, Buprenorphine, Buprenorphine and naloxone, Disulfiram, ethadone, Naltrexone, Varenicline) (1SR, 18 RCTs)  Disulfiram (12)  Dopamine Agonists (24)  Other pharmacotherapies (19)  Pharmacotherapies for comorbid opioid use disorder (6 SRs, 14 RCTs) | RCTs of adults with cocaine use disorder comparing pharmacotherapies. Studies involving participants with comorbid psychotic spectrum or bipolar disorders were excluded. | Primary outcomes: sustained abstinence, cocaine use, treatment retention, serious adverse events and dropout.  Increased abstinence found with bupropion, topiramate, and psychostimulants. Antipsychotics improved treatment retention. |
| *Torrens et al. 2005* | 19 RCTs, N = 1180  Cocaine dependent participants with and without comorbid depression, without other Axis I psychiatric diagnoses.  duration: mean 9.9 wks (range 2-13 wks) | Without comorbid Depression:  Desipramine vs placebo (7)  Gepirone vs placebo (1)  Fluoxetine vs placebo (3)  Imipramine vs placebo (1)  Ritanserine vs placebo (1)  Bupropion vs placebo (1)  With Comorbid Depression:  Desipramine or Amantadine vs placebo (1)  Imipramine vs placebo (1)  Fluoxetine vs placebo (1)  Desipramine vs placebo (1) | RCTs assessing the use of antidepressants in cocaine dependent participants with or without comorbid depression. RCTs were excluded that had a Jada quality score of 2 or less. Studies that included patients with other psychiatric comorbidities were excluded. | *Cocaine use without comorbid depression:* No significant results supporting antidepressants for cocaine dependence. Weak but significant reduction in cocaine use for other antidepressants other than SSRIs.  *Cocaine use with comorbid depression:* No significant decrease in cocaine consumption. |
| *Chan et al. 2019* | 34 RCTs examining 17 different drugs.  Participants were adults with methamphetamine/amphetamine use disorder. | Antidepressants (1 SR incl. 21 RCTs, 3 RCTs)  Antipsychotics (2)  Muscle relaxants, Anticonvulsants (2)  Opioid antagonists (4)  Varenicline (1)  Atomoxetine (1)  Other pharmacotherapies (4)  Psychostimulants (1 SR incl. 21 RCTs) | RCTs of adults with MA/A use disorder examining pharmacotherapy (head to head) or placebo or psychotherapy.  Studies were excluded that did not perform UDS once per week. | Primary outcome: abstinence, defined as 3 or more consecutive weeks with negative urine drug screens, overall use (proportion of negative UDS), and treatment retention.  Methylphenidate may reduce MA/A use.  Antidepressants, anticonvulsants, antipsychotics, naltrexone, varenicline and atomoxetine had either low-strength or insufficient evidence of no effect.  Subgroups: MA/A severity at baseline, methamphetamine-negative UDS at randomization, gender, comorbid or life-time alcohol use disorder, comorbid ADHD, comorbid depression, HIV status.  Subgroup analyses limited by low quality data. Bupropion and Topiramate demonstrated increased effect in certain subgroups (decreased MA/A severity, males and negative UDS at baseline, respectively) |
| *Pani et al. 2010* | 7 studies, 492 participants  Cocaine dependence  68.4% male  mean age 38 years  Studies included participants with additional substance use diagnoses, and those with comorbid mental health conditions.  Mean duration of trials: 12 weeks (range 11-12 weeks) | Disulfiram vs placebo (4)  Disulfiram vs naltrexone (3)  Disulfiram vs no pharmacological treatment (2) | RCTs and controlled clinical trials focusing on use of disulfiram for cocaine dependence were included.  People under 18 years old and pregnant women were excluded. | Primary outcomes: dropout, side effects, use of primary substance of abuse, cocaine use at follow-up.  One study found statistically significant difference in cocaine use favoring Disulfiram, compared to no pharmacological treatment.  Compared to placebo, no benefit was found for participant dropout. One study found a reduction in cocaine use favoring Disulfiram.  No significant difference in dropout or cocaine use compared to Naltrexone. |
| *Minozzi et al. 2015* | 24 studies, N = 2147  Cocaine Dependence  82% male  mean age 37  Participants with additional substance use diagnoses and psychiatric comorbidities were included.  Mean duration of included trials: 7 weeks (range 1.5-16 weeks) | Dopamine agonists (# trials)  Amantadine vs placebo (10)  Bromocriptine vs placebo (5)  L-dopa/carbidopa vs placebo (6)  Pergolide vs placebo (2)  Cabergoline vs placebo (1)  Hydergine vs placebo (1)  Pramipexole vs placebo (1)  Amantadine vs desipramine (4)  Amantadine vs fluoxetine (1)  Amantadine vs propranolol (1) | RCTs and controlled clinical trials focused on the use of dopamine agonists for cocaine misuse.  People under 18 years old and pregnant women were excluded. | Primary outcomes: Dropouts, adverse effects, abstinence (self-reported or negative UDS), and abstinence at follow-up.  No difference in dropout, abstinence, severity of dependence or adverse events.  Low quality evidence that antidepressants performed better than Amantadine for abstinence, no differences found for dropout or adverse events |
| *Indave et al. 2016* | 14 RCTs, N = 719  Cocaine dependence  66.3% male  mean age: 41.5 years  Participants with additional substance use diagnoses, and psychiatric comorbidities were included. | Antipsychotics:  Risperidone vs placebo (4)  Olanzapine vs placebo (3)  Quetiapine vs placebo (2)  Lamotrigine vs placebo (1)  Reserpine vs placebo (1)  Olanzapine vs haloperidol (1)  Olanzapine vs risperidone (1)  Aripiprazole vs ropirinol (1) | All randomized controlled trials and controlled clinical trials focused on use of any antipsychotic medication for cocaine dependence.  People under 18 years old and pregnant women were excluded. | Primary outcomes: dropout, number of participants using cocaine during treatment, continuous abstinence, side effects, craving.  Compared to placebo, antipsychotics reduced dropout. No significant benefit found for remaining outcomes studied.  Quetiapine showed reduction in cocaine use (one study). |
| *Alvarez et al. 2013* | 12 RCTs, N = 681  Cocaine abuse or dependence  Mean % male: 78%  Mean age: 39  Mean % African-American: 62.2%  Participants with additional substance use diagnoses, and with comorbid psychiatric diagnoses were included.  Duration of treatment range 6-26 weeks | Antipsychotics:  Risperidone vs. placebo (4)  Olanzapine vs. placebo (3)  Reserpine vs. placebo (2)  Ritanserin vs. placebo (2)  Quetiapine vs. placebo (1) | Studies included participants using cocaine only, were double-blind, randomized controlled, with parallel group design. | Primary outcomes: cocaine use, treatment retention, craving, addiction severity index.  Antipsychotics did not significantly reduce cocaine use (quantified by urine sample and self-reported cocaine use).  Antipsychotics did not improve treatment retention. There was a small but statistically significant reduction in dropout with the use of risperidone compared to placebo.  Reduced craving was reported in the treatment group. |
| *Kishi et al. 2013* | 14 studies, N = 741  Participants with primary cocaine dependence (10 studies) or methamphetamine/amphetamine dependence (4 studies) without psychiatric comorbidities.  Average age: 40.5 (cocaine dependence), 34.85 (amphetamine dependence)  78.1% male (cocaine); 83.6% male (amphetamine)  Average % white: 38.3% | Antipsychotics  Primary cocaine dependence:  Risperidone vs placebo (5)  Olanzapine vs placebo (3)  Reserpine vs placebo (2)  Amphetamine/methamphetamine dependence:  Aripiprazole vs. placebo (4) | RCTs of antipsychotics lasting at least 2 weeks.  Patients with comorbid psychiatric conditions were excluded. | Primary outcomes: Cocaine use and abstinence (negative screens throughout treatment period or at last visit).  Antipsychotics were not superior to placebo for the outcome of cocaine use. Data for methamphetamine use was not analyzable.  Antipsychotics were not superior to placebo in the outcome of abstinence in both cocaine and methamphetamine subgroups. |
| *Minozzi et al. 2015* | 20 studies, N = 2068  Cocaine dependent  Participants with additional substance use diagnoses and psychiatric comorbidities were included.  Mean trial duration 11.8 weeks (range 8-24 weeks)  84.5% smoked crack cocaine, 10.6% intranasal, 6.6% IV (13 studies reported this information) | Anticonvulsants vs Placebo:  Carbamazepine (6)  Gabapentin (3)  Lamotrigine (2)  Phenytoin (1)  Tiagabine(3)  Topiramate (5)  Vigabatrin (2)  Carbaazepine vs desipramine (1) | People under 18 years old and pregnant women were excluded. | Primary outcomes: dropout, use of primary substance (reported or positive UDS), side effects  When anticonvulsants were compared to placebo, no significant difference was reported for any of the efficacy and safety measures, including dropouts, cocaine use (defined as patient reported cocaine use during treatment or cocaine positive urine samples), cravings, severity of dependence, depression or anxiety.  Subgroup analyses: single types of anticonvulsants versus placebo. No significant difference found for any of the primary outcomes. |
| *Alvarez et al. 2010* | 15 RCTs, N = 1,236  Cocaine dependence (DSM-III, IV)  Participants with additional substance use diagnoses and comorbid psychiatric diagnoses were included.  Median age 36 range 20-60  80% male  72% African American  Minimum duration 8 weeks | Anticonvulsants vs placebo:  Carbamazepine (7)  Phenytoin (1)  Valproic acid (1)  Tiagabine (3)  Tiagabine and Gabapentin (1)  Gabapentin and Lamotrigine (1)  Topiramate (1) | Randomized, controlled by placebo and parallel group, double blind clinical trials designed for clinical testing  Cocaine use based on urine screen | 2 outcome measures: retention in treatment, subsequent cocaine use (urinalysis)  No improvement in treatment retention.  No reduction in cocaine use, except in one study (Topiramate vs placebo). |
| *Singh et al. 2015* | 5 Studies, N = 518  Mean age: 42.9 years  % Male: 71%  Participants with cocaine dependence or use disorder  Mean duration of trials: 13 weeks (range 12-18) | Topiramate vs placebo (4)  Topiramate vs no medication (1) | Peer reviewed RCTs  Participants met criteria for cocaine dependence or use disorder without concurrent disorder | Primary outcomes: treatment retention, continuous abstinence and cocaine use (number of weeks of cocaine free urine, or self-reported cocaine use days).  No significant reduction in dropout.  Topiramate was associated with significant increase in continuous abstinence – analyzed from 2/5 studies  Improvement in subjective craving scores in one study |
| *Castells et al. 2016* | 26 studies, N = 2366  Participants with cocaine dependence (DSM cocaine abuse or dependence). Studies included participants with comorbid psychiatric and additional substance use diagnoses.  Mean % men: 75%  Mean age: 39.6  % African-American: 47.6%  % White: 39.3%  Mean trial duration 12.6 weeks (range 6 – 24 weeks)  Route of use: 60.8% inhalation | Psychostimulants vs. placebo:  Bupropion (3)  Dexamphetamine (4)  Lisdexamfetamine (1)  Methylphenidate (4)  Modafinil (8)  Mazindol (4)  Methamphetamine (1) Mixed amphetamine salts (1)  Selegiline (1) | Randomized parallel group placebo-controlled clinical trials | Primary outcomes: Reduction in use (proportion of negative urinalysis), sustained cocaine abstinence, retention in treatment.  Very low quality evidence that psychostimulants improve cocaine abstinence.  No significant reduction in cocaine use. Psychostimulants did not increase retention in treatment.  One study reported a significant difference favoring psychostimulants for participant-rated addiction severity and participant rated addiction severity improvement.  Two studies assessed comorbid heroin use and reported improvement in sustained heroin abstinence, but no significant difference in heroin use.  Subgroup analyses: Type of CNS stimulant, clinical definition of cocaine use disorder, comorbidities, study quality, type of administered scales, single site vs. multisite, funding.  Modafinil was superior to placebo in reducing cocaine use. Bupropion, dexamphetamine and mixed amphetamine salts improved abstinence. Dexamphetamine improved heroin abstinence in comorbid opioid use disorder. Mixed amphetamine salts improved ADHD symptom severity. Psychostimulants reduced cocaine use and increased cocaine abstinence in studies in which ADHD was not an inclusion criterion. |
| *Perez-Mana et al. 2013* | 11 studies, N = 791  Participants with amphetamine abuse or dependence.  Participants with additional substance use diagnoses, and psychiatric comorbidities were included.  64.9% male  mean age 35.9  9 studies: methamphetamine-dependent  two studies: amphetamine-dependent participants | Psychostimulant vs placebo:  Dexamphetamine (2)  Bupropion (3)  Methylphenidate (2)  Modafinil (4) | Studies lasting less than 4 weeks were excluded. | Primary outcomes: Amphetamine use (mean negative UA across study, amphetamine concentration in hair), sustained amphetamine abstinence.  No significant difference in amphetamine use or sustained abstinence.  No significantly significant results were reported for any of the secondary outcomes including self-reported amphetamine use, retention in treatment, craving, and safety outcomes.  Subgroup analyses: Type of psychostimulant, type of amphetamine dependence, study length, comorbidities, published vs unpublished data, risk of bias. No difference in efficacy, safety, and retention between psychostimulants and placebo in any subgroup analyses. |
| *Bhatt et al. 2016* | 17 studies, N = 1387  Participants with methamphetamine or amphetamine dependence.  Mean % male: 70.5%  One trial in adolescents.  Remaining trials adults age 18-65  One trial specifically included men who have sex with men  Mean trial duration: 14.9 weeks (range 8 – 24) | Psychostimulants vs placebo:  Modafinil (3)  Bupropion (6)  Methylphenidate (6)  Dexamphetamine/dextroamphetamine (2) | RCTs comparing psychostimulants to placebo.  Non-randomized and quasi-randomized studies were excluded.  Participants > 14 years of age were included. | Primary outcomes: abstinence (methamphetamine-free urine screens during the final two weeks of the trial), retention in treatment and safety.  No significant effect on abstinence. (5 studies). No significant effect on treatment retention (14 studies). No significant difference in adverse events (3 studies)  Subgroup analyses: amphetamine vs methamphetamine use disorder, type of psychostimulant, frequency of amphetamine and methamphetamine use, age of participants and duration of treatment.  Subgroup of > 12 weeks of treatment showed increased retention compared to shorter duration. All other subgroup analyses found no significant differences. |
| *Perez-Mana et al. 2011* | 29 studies, N = 2,467  Cocaine (86.3%)or amphetamine (13.87%) dependence  26.8% had comorbid opioid dependence  Duration: range 6-26.7 wks | Indirect Dopamine Agonists (IDAs)  Bupropion (5)  Dexamphetamine (3)  Disulfiram (4)  Levodopa (2)  Mazindol (4)  Methamphetamine (1)  Methylphenidate (4)  Modafinil (3)  Selegiline (1) | RCTs assessing efficacy of IDAs in psychostimulant dependence. IDA was defined as ‘any drug that, irrespective of its mechanism of action, increased extracellular dopamine’. | Primary outcome: psychostimulant abstinence (drug-free UA)  Significant increase in stimulant abstinence.  No increase in treatment retention.  Efficacy was larger in comorbid heroin dependent participants.  Dexamphetamine and bupropion were the only interventions that had a statistically significant increase in abstinence compared to placebo. |
| *Sangroula et al. 2017* | 11 studies, N = 896  Participants with cocaine dependence (DSM-IV) without comorbid psychiatric conditions. Only nicotine (n =5), marijuana (n=3) ad alcohol (n=1) comorbid substance use disorders were included.  Mean age: 42.4 years  74.3% male  69% African-American  32.6% White  mean duration 6.7 weeks | Modafinil vs. placebo | Blinded RCTs with greater than 20 subjects meeting criteria for cocaine dependence or use disorder  Comorbid psychiatric conditions were excluded except nicotine, marijuana, and alcohol. | Primary outcomes: Treatment retention and abstinence (defined as number of subjects who became abstinence from using cocaine determined by UDS or self-report).  Modafinil was not superior to placebo in the outcomes of treatment retention, or abstinence.  Modafinil was superior to placebo in the outcome of cocaine non-use days and number of negative urine samples.  Subgroup analysis showed significant improvement in cocaine abstinence in trials conducted in the United States (10 studies) |
| *Dursteler et al. 2015* | 5 RCTs, N = 363  Cocaine dependence  73.5% male  3/5 trials (252 participants) included only adults with ADHD | Methylphenidate vs placebo | Double-blind, randomized, and placebo-controlled trials assessing efficacy of MPH as replacement therapy for cocaine dependence | No significant reduction in cocaine use with MPH treatment. |
| *Castells et al. 2009* | 37 studies, N = 3,029  Participants with dual opioid and cocaine dependence  Mean age range 32.0-42.6  63% men  55% Caucasian  all patients with heroin dependence, 93% had comorbid cocaine dependence | Opioid Maintenance Therapy (6)  No placebo group. 3 studies assessed high vs low dose OMT  Adjunctive interventions to OMT (34) | RCTs that assessed efficacy of OMT and OMT + adjunctive therapies in opiate dependent patients with comorbid cocaine use disorder. | Primary outcomes: heroin abstinence and cocaine abstinence.    High doses of OMT were more effective for sustained heroin abstinence but had no effect on cocaine abstinence.  Methadone showed greater improvement than buprenorphine in cocaine abstinence, at equivalent doses.  No dose response was observed for methadone treatment in the outcome of cocaine abstinence. |
| *Echevarria et al. 2017* | 6 clinical trials  192 human participants  Participants with cocaine dependence  duration: mean 2.7 weeks (range: single dose – 8 weeks) | N-Acetylcysteine (NAC) vs placebo (3)  NAC at 3 doses (1)  NAC vs. baclofen (1)  NAC vs no medication | Review included both human trials and animal studies. Only human trials were included in our review. 3 studies were double-blind placebo controlled, one was pre-clinical single-blind trial and one was an open-label clinical trial. | Mixed results reported across six human trials. One trial found a reduction in interest in cocaine-related stimuli, but no significant reduction in craving. One trial found a significant reduction in cocaine use days and dollars spent on cocaine. Reported craving was significantly reduced in the NAC group compared to baclofen. One study found a significant reduction in the glutamate/creatinine ration in the left anterior cingulate cortex on magnetic resonance spectroscopy. Abstinence, measured by Benzoylecgonine levels was not significantly affected (one trial), but subgroup analyses showed increased time to relapse in already abstinent patients. |

*RCT, Randomized controlled trial; CBT, Cognitive behavioral therapy; CM, Contingency management; TAU, Treatment as usual; CRA, Community reinforcement approach; DT, Day treatment; MA/A, Methamphetamine/Amphetamine; SR, Systematic review; ADHD, Attention deficit hyperactivity disorder; UDS, Urine drug screen; NAC, n-acetylcysteine*
