# Supplementary material for: Treatment of stimulant use disorder: A systematic review of reviews
Source: PLoS One. 2020 Jun 18;15(6):e0234809. doi: 10.1371/journal.pone.0234809 (PMC7302911; doi:10.1371/journal.pone.0234809)
Supplement: Attachment — Submitted filename: Response to Reviewers.docx [file pone.0234809.s005.docx]

**RESPONSE TO REVIEWER’S COMMENTS:**

********Any new text in the revised manuscript has been noted below in* ***bold font.***

**Editor:**

1. In accordance with PLOS ONE’s style requirements, we have updated the manuscript title and references, as well as the file names.

2. Description of funding information has been removed from the manuscript in accordance with PLOS requirements. We would like to update our Funding Statement as follows:

"This research was undertaken, in part, thanks to funding from a MSFHR/St. Paul’s Foundation Scholar Award which supports Dr. Nadia Fairbairn. Evan Wood is supported by a Tier 1 Canada Research Chair in Addiction Medicine. Kanna Hayashi is supported by a CIHR New Investigator Award (MSH-141971), a Michael Smith Foundation for Health Research (MSFHR) Scholar Award, and the St. Paul’s Hospital Foundation. Dr. Seonaid Nolan is supported by the MSFHR and the University of British Columbia’s Steven Diamond Professorship in Addiction Care Innovation. Rod Knight is supported by a Scholar Award from MSFHR. A European Commission grant (701698) and Canadian Institutes of Health Research (671 397968, 422332) grants support Dr. Klimas."

3. As requested for PLOS submissions, we have updated ORCID ID information and validated it in Editorial Manager.

4. Supporting Information files have been included at the end of the manuscript, and in-text citations have been updated in accordance with PLOS guidelines.

5. Appendix S1 and S2 referenced on pages 4 and 5 of the manuscript have been updated and uploaded as Supporting Information.

**Reviewer #1:**

1. We thank Reviewer #1 for identifying discrepancies between the overview of included systematic reviews and statements of evidence presented in Table 2 and the Abstract and Conclusion sections of the manuscript. Discrepancies were correctly identified by the Reviewer with respect to psychostimulants, n-acetylcysteine (NAC) and opioid agonist therapy (OAT) interventions. We have made several changes to ensure the evidence statements in Table 2 align with the Abstract:
i. The summary of evidence for psychostimulants is also now aligned in the Abstract section with Table 2, which found insufficient evidence to support or discount its use.
ii. For NAC, we have removed the statement in the Abstract regarding positive results for NAC to align with the evidence statement in Table 2 which found insufficient evidence to support or discount its use.
iii. Regarding the evidence statement for OAT, the study reviewers re-appraised the included review by Castells et al. (2009) and have adjusted the evidence statement to be insufficient to support or discount its use (Table 2). The evidence statement for OAT was previously categorized as tentative evidence to support the effectiveness of the intervention. The study reviewers decided to adjust the evidence statement for OAT because the positive benefits for OAT in previous reviews are limited to a select subgroup of people with dual heroin-cocaine dependence, and only comparative evidence between OAT therapies were found, with no comparisons between OAT and placebo. We thank Reviewer #1 for bringing this discrepancy to our attention.

Accordingly, we have revised the results section of the Abstract to align with the evidence statements presented in Table 2, as follows:

**“Psychostimulant, n-acetylcysteine, opioid agonist therapy, disulfiram and antidepressant pharmacological interventions were found to have insufficient evidence to support or discount their use.” *(Abstract, page 2)***

We have also revised the conclusion statement regarding psychostimulants in the Abstract as follows:

**“Although evidence to date is insufficient to support the clinical use of psychostimulants, our results demonstrate potential for future research in this area.” *(Abstract, page 2)***

2. We appreciate the suggestion from Reviewer #1 to align the presentation of evidence statements in Table 2 with the Results section of the manuscript to improve ease of interpretation of data for the reader. We have now arranged both the Results section and Table 2 by cocaine use first, followed by amphetamine/methamphetamine use. Additionally, core reviews are reported before supplementary reviews in the text. Table 2 now aligns with the order of presentation of interventions in the Results section of the manuscript and should be easier to follow for the reader.
a. Reviewer #1 also brought forward the names of several review papers on treatment of stimulant use disorder for further information regarding our decision not to include them in the current study.
i. Regarding the De Crescenzo et al. (2018) article, this review was a comparative efficacy and acceptability study of a range of psychosocial interventions for individuals with cocaine and amphetamine addiction. This study was initially identified using our search strategy and was excluded based on our criteria not to include reviews where the intervention was not clearly defined, due to the fact that this study primarily focused on comparative effectiveness of psychosocial treatments used in combination (*Methods section, page 5*). However, the reviewers scrutinized this study again and determined that it is possible to separate results by intervention in this study as per our inclusion criteria for the study, and have thus opted to include it in our review. We thank the Reviewer for their helpful feedback and feel the manuscript is strengthened by inclusion of this paper in our study. We have updated Table 2 accordingly, and have added additional text in the manuscript regarding findings from this review *(Results, pages 15-17):*

**“De Crescenzo et al. included 50 RCTs evaluating 12 psychosocial interventions for the treatment of stimulant use disorder, including 15 RCTs that compared CM alone to treatment as usual (TAU). A significant benefit was found for the outcomes of abstinence at 12 weeks (Odd’s Ratio [OR] 2.29, 95% Confidence Interval [CI] 1.62, 3.24), abstinence at the end of treatment (OR 2.22, 95% CI 1.59, 3.10), dropout at 12 weeks (OR 1.39, 95% CI 1.09, 1.78), and dropout at the end of treatment (OR 1.41, 95% CI 1.10, 1.82), but the effect was not sustained at longest follow up (OR 1.10, 95% CI 0.83, 1.46) (15).” *(Results, page 15)***

**“De Crescenzo et al. found CM alone as well as CM in combination with CBT to be superior to CBT alone in the outcome of abstinence at the end of treatment (OR 1.88, 95% CI 1.52, 2.85, and 2.08, 95% CI 1.28, 3.33, respectively). Of note CM compared to CBT at 12 weeks of treatment, and at longest follow up were not reported (15).” *(Results, page 15, 16)***

**“De Crescenzo et al. found the combination of CM with CRA to be the only intervention that increased the rate of abstinence at the end of treatment (OR 2.84, 95% CI 1.24, 6.51), at 12 week follow up (OR 7.60, 95% CI 2.03, 28.37) and at longest follow up (OR 3.08, 95% CI 1.33, 7.17). They found this combination to be more effective than CBT (OR 2.44, 95% CI 1.02, 5.88), non-contingent rewards (OR 3.31, 95% CI 1.32, 8.28), and 12-step programme plus non-contingent rewards (OR 4.07, 95% CI 1.13, 14.69). CM with CRA also demonstrated significant benefit in the outcome of participant dropout when compared to TAU both at 12 weeks (OR 3.92, P < 0.001) and at the end of treatment (OR 3.63, P < 0.001) (15).” *(Results, page 16, 17)***

**“The review by De Crescenzo et al. included seven RCTs comparing CBT to TAU. A significant benefit was found in the outcomes of dropout at 12 weeks [OR 1.42, 95% CI 1.05, 1.93], and dropout at the end of treatment [OR 1.47, 95% CI 1.08, 2.00]. However, no significant benefit was identified for abstinence at 12 weeks, at the end of treatment, or at longest follow up (15).” *(Results, page 18)***

ii. The Tardelli et al. (2018) paper provided an interesting review on contingency management (CM) combined with pharmacotherapy. We reviewed this paper again as it was identified as part of our search strategy and not included as it did not meet our inclusion criteria. It was a qualitative review rather than a systematic review and meta-analysis that did not meet our inclusion criteria due to its study design.
iii. Similar to the Tardelli et al. (2018) paper, the Ballester et al. (2017) paper was reviewed again and we determined it was properly excluded from our study as it was a qualitative review and did not meet our inclusion criteria due to its study design.

b.	We appreciate Reviewer #1 indicating that the Schumacher et al. (2007) reference was missing from the References list; this has been updated accordingly.

c.	We appreciate the suggestion from Reviewer #1 to include the reference number for each citation in Table 2 and have updated accordingly.

d.	We agree with Reviewer #1 that Table 2 could be re-organized to improve usefulness for the reader. As indicated above, we have now arranged Table 2 by cocaine use first, followed by amphetamine/methamphetamine use. We have additionally placed core reviews before supplementary reviews in the Table. This layout now also aligns with the organization of the Results section of the manuscript.

e.	Please see comment above regarding how we have improved the layout of Table 2 and aligned it with the Results section of the manuscript.

f.	We appreciate the excellent observation from Reviewer #1 regarding the Discussion section, where we discussed some but not all evidence statements where we found insufficient evidence to support or discount the intervention. All of the interventions appraised as insufficient to support or discount their use are now included in the Discussion section, with recommendations for future research. Additional text reads as follows:

**“CBT has been studied both alone and in combination with other psychotherapy interventions for treatment of stimulant use disorder, with some positive results. De Crescenzo et al. found a reduction in participant dropout with CBT alone, however when combined with CM, CBT was found to have a more pronounced effect, including in the outcome of participant abstinence (15). The available data for CBT in the treatment of stimulant disorder is limited, and more research is warranted to determine its clinical utility, focusing on a potentially more sustained effect when employed complementarily with contingency management psychosocial interventions, or pharmacologic treatment options.”   *(Discussion, page 34, 35)***

**“Similarly, for disulfiram, data supporting its clinical utility in treatment of stimulant use disorder is limited. The review by Pani et al. found positive results in reduction in use for one of four included RCTs (28). However, concerns regarding the safety of disulfiram, particularly in concurrent alcohol users, may limit its potential for future research (58).” *(Discussion, page 35)***

**“Current data does not support the use of antidepressants for treatment of cocaine use disorder, although with regards to amphetamine use there is insufficient evidence to discount its use. Due to predominantly negative results for the use of antidepressants for treatment of cocaine use disorder, it may be possible to extrapolate from this data for the treatment of amphetamine use disorder (24, 25, 27). However, due to the unique mechanisms of action on presynaptic monoamine reuptake transporters, further research focused on the utility of antidepressants for amphetamine-type stimulants may be warranted.” *(Discussion, page 35,36)***

3. We appreciate the suggestions for reorganizing the text, especially the psychostimulant section. As suggested by the Reviewer and detailed above, the body of Results section in the manuscript is now organized by cocaine use first, followed by amphetamine or methamphetamine use. Studies classified as core reviews are also presented before supplementary reviews for each intervention type.

4. We thank the Reviewer for pointing out that not all citations in Table 2 are found in the Appendix. The appendix (now entitled Appendix S3) has been updated with the remaining citations, and has additionally been reorganized to align with the Table 2 presentation of evidence statements.

Minor Comments:

5.	We appreciate the observation from the Review that line 94-96 did not fit well. We have revised this sentence to improve clarity:

**“Of those accessing publicly funded treatment for substance use disorder in the United States, less than one in five individuals (17.8%) are doing so for cocaine or other stimulant treatment (8).” *(Introduction, page 3)***

6.	We thank the Reviewer for identifying the use of DSM-V. Text now reads DSM-**5**. (Materials and methods page 5)

7.	We agree with the Reviewer that the following line disrupted the flow of the text and it has been moved to the subsequent paragraph:

**“Methods of searching and assessing primary literature, appropriateness of outcomes measured and conclusions, recognition of biases and steps taken to limit those biases, as well as the use of the PRISMA guidelines for systematic reviews were assessed.” *(Materials and methods, page 8)***

8.	We thank the Reviewer for the suggestion to add the number of discrepancies that were resolved by a third reviewer. This has been added to the text as follows:

**“There were two discrepancies which were resolved by a third independent reviewer (SN).” *(Materials and methods, page 8)***

9.	We thank the Reviewer for identifying that Schumacher et al. was not included in the references. This has been updated. Schumacher et al. is a meta-analysis of four primary RCTs and met our inclusion criteria as supplementary evidence.

10.	We appreciate the observation by the Reviewer that the references were not aligned with the text. This has been updated accordingly.

11.	We agree with the suggestion from the Reviewer to change “were” to **“was”** (Results, page 18)

12.	We appreciate the suggestion from the Reviewer to adjust the abbreviations used in the text. “SMD” was not spelled out at first use and has now been updated accordingly. “WMD” is spelled out at first use on page 21. “SMD” has been updated at first use as **“standardized mean difference (SMD)” *(Results, page 27)***

13. We appreciate the Reviewer identifying the discrepancy in citation #55 and the citations have been updated accordingly.

14. We thank the Reviewer for identifying that due to multiple reviews by Castells et al., it is unclear which review is being referred to. This has been updated in the text to include the date for clarity:

**“Castells et al. (2016)” *(Discussion, page 34)***

15.	We thank the Reviewer for identifying inconsistencies in the formatting of the references. This has been updated accordingly.

**Reviewer #2:**

1. We appreciate the suggestion by the Reviewer to further discuss the definition of ‘recovery’ as it applies to standardized outcome measures. We have added to the Discussion section a description of changing views on how to define outcome measures in substance use research that goes beyond measures of abstinence (Discussion, page 36,37):

“Another limitation of meta-analyses to date is the lack of standardized outcomes, making pooling of data difficult. Without evidence-based, standardized clinical trial outcomes for the treatment of stimulant use disorder, it will remain difficult to pool data and provide strong clinical recommendations. **Long-term cessation of use has traditionally been the primary goal of substance use treatment and abstinence measures have been the most commonly implemented standard outcomes in randomized controlled trials (61). However, the definition of recovery may vary based on individual patient goals, by feasibility within the study time period, and may not always include abstinence (62). In 2015, the Analgesic, Anesthetic, and Addiction Clinical Trial Translations, Innovations, Opportunities, and Networks (ACTTION) group made recommendations for meaningful indicators of treatment success in future clinical trials on stimulant use, including a focus on the validation of patient reported outcome measures of functioning and the exploration of patterns of stimulant abstinence that may be associated with physical and/or psychosocial benefits (62).** Future study may benefit from patient-oriented outcome measures, including psychosocial parameters such as improved social functioning, employment and acquisition of adequate housing.” ***(Discussion, page 36, 37)***

2. We thank Reviewer #2 for the important suggestion to include details regarding subgroup effects of the interventions. We have added another table as supplementary material (Table S1) that provides more detailed information on population characteristics of each study, as well as any subgroup analyses that were reported. The following text was also added to the Discussion section: *(Discussion, page 37, 38)*

**“It is important to note that the variability in treatment response among population subgroups is poorly addressed in the literature. Given the extensive variability in individual response to treatment, it is possible that an intervention appraised as ‘sufficient evidence to discount the effectiveness’ or ‘insufficient evidence to support or discount its effectiveness’ may have significant benefits for some patients or patient subgroups. In studies that performed subgroup analyses, including but not limited to age, sex, severity of substance use disorder and comorbid substance use disorders, meaningful results were limited. Future research would benefit from identifying those subgroups that may be more likely to benefit from certain interventions.” *(Discussion, page 37)***

**“Subgroup analyses by Bhatt et al. demonstrated increased retention in treatments of longer duration (> 12 weeks) (37). Stratification by treatment duration moving forward may aid in identifying optimal and minimum effective treatment durations. Castells et al. (2016) found that psychostimulants increased abstinence and reduced cocaine use in those studies in which Attention Deficit Hyperactivity Disorder (ADHD) was not an inclusion criterion, which may be an important replicate moving forward (36).” *(Discussion, page 37, 38)***

3. We thank Reviewer #2 for raising the issue of people who use polysubstances. This is an important point to identify, as polysubstance use was the norm in the studies reviewed here. Table S1 has identified which reviews include participants with concurrent additional substance use disorders. We have added a section in the discussion regarding polysubstance use as follows:

**“Finally, polysubstance use is common (67, 68). Recent data demonstrates a rise in concurrent amphetamine and opioid use, and the role of stimulant use in the overdose epidemic remains poorly defined (69)*.* Several of the included reviews identified that many participants suffered from polysubstance use disorder, however the effect of polysubstance use on treatment efficacy was rarely addressed. Future research would benefit from identifying the efficacy of interventions for stimulant use disorder specifically in the context of polysubstance use.” *(Discussion, page 38)***

General Comments:

1. Thank you for identifying the outdated World Drug Report citation. These statistics have been updated from the 2019 World Drug Report.
2. References have been corrected.
